# Diversity of Menstrual Cycle, Formation of Decidual Cells, and Lack of Endometrial Glands in Spiny Mouse

**DOI:** 10.3390/biology14101365

**Published:** 2025-10-05

**Authors:** Roman Eremichev, Nina Nikolaeva, Mikhail Khandokhin, Roman Tsvetcov, Natalya Alexandrushkina, Alena Shilova, Vladimir Popov, Pavel Makarevich

**Affiliations:** 1 Centre for Regenerative Medicine, Lomonosov Moscow State University, 119192 Moscow, Russia; eremichevry@my.msu.ru (R.E.); nd.nikolaeva@mail.ru (N.N.); khandokhinmm@my.msu.ru (M.K.); tsvetcovroman01@yandex.ru (R.T.); alexandrushkinana@my.msu.ru (N.A.); galiantus@gmail.com (V.P.); 2 Faculty of Medicine, Lomonosov Moscow State University, 119192 Moscow, Russia; ladybird-a@yandex.ru

**Keywords:** *Acomys*, spiny mouse, menstruation, decidualization, nerves, uterine glands, spiral arteries

## Abstract

**Simple Summary:**

Models of endometrium turnover via menstrual cycle are limited and recent discovery of *Acomys* genus spiny mouse that possesses this physiological feature provided a valuable tool for researchers. In our spiny mice colony we failed to detect typical mensutral bleeding while we encountered decidual cells and lack of endometrial glands which contrasted to CD1 laboratory mice. Overall, we highlight importance of *Acomys* identification between colonies and their potential application to study the role of stromal cells in endometrium renewal and uterine cycle regulation.

**Abstract:**

Recent discovery of menstruation in the Egyptian spiny mouse (*Acomys cahirinus)* highlighted this species as a feasible model for the study of menstrual cycle physiology. However, reports on active menstrual bleeding in this animal were contradictory, so we set out to reproduce major findings in the field. Using vaginal smear microscopy and occult blood assay, we have failed to detect menstrual bleeding in spiny mice from our colony at Lomonosov Moscow State University. Otherwise, we demonstrated appearance of well-defined decidual cells during the late secretory phase of the cycle that correlated with an increase in serum progesterone. Comparing the uteri of spiny mice from our colony vs. CD1 strain laboratory mice housed in the same animal unit, we have found several noteworthy features: (1) absence of endometrial glands, (2) higher volume of nerve fibers in the endometrium, and (3) spiral-like arteries in myometrium. Taking results of other groups into account, our results highlight putative diversity of menstrual cycles in spiny mice from different colonies and demonstrate important differences in uterus structure compared to *M. musculus*.

## 1. Introduction

Endometrium (uterine mucosa) plays a pivotal role in human reproduction: its normal function is closely related to menstruation defined as a periodic (monthly) bleeding from the genital tract in women. During menstruation, the endometrium exfoliates, undergoes rapid healing, and in later phases of menstrual cycle completely restores resulting in full-thickness regeneration of this functional layer.

However, menstrual cycle is not as common among species as one may expect. Indeed, among placental animals, the majority demonstrate an estrous cycle in which ovulation is regulated by sex hormones, male pheromones, and seasonal changes, but excludes menstruation as defined above [1]. Among approximately 6000 identified mammals, less than 2% adopt a reproductive strategy of cyclic shedding and restoration of the endometrium.

In menstruating animals, endometrium undergoes regular shedding followed by vaginal bleeding. These events are preceded by decidualization of the endometrial mucosa, including phenotype changes of stromal cells to secretory decidual cells. The latter is a prerequisite for embryo implantation involving disruption of epithelium and trophoblast invasion to the endometrial layer.

Decidualized endometrium provides a physiological interface between the embryo and the maternal organism. It is rich in capillaries, decidual, and regulatory immune cells that contribute to the normal and timely development of the fetus. It is noteworthy that in non-menstruating mammals, decidualization is triggered by embryo implantation to the uterine mucosa. Described sequence is contrasting to events in menstruating mammals in which decidualization is driven by hormones (predominantly progesterone) prior to and regardless of fertilization [2]. If pregnancy does not occur, the decidualized endometrium is rejected by shedding during menstruation.

Generally, menstruation is an important reproductive trait characteristic exclusively for mammals that exhibit spontaneous decidualization. From this point of view, menstruation is a periodic release of blood and pieces of decidualized endometrium induced by decline of blood progesterone levels [3,4].

The majority of identified menstruating species belong to the order of *Primates,* while few exceptions are known, including several bat species and the elephant shrew [1]. Recent discovery of menstruation in the Egyptian spiny mouse (*Acomys cahirinus*) places this species within a small group of menstruating mammals. Taking into account the general similarities between the house and laboratory mouse (*Mus musculus*) including anatomy, husbandry, and ease of handling, the spiny mouse is considered as a model species for the study of menstrual cycle physiology. This provides researchers with an accessible model (especially compared to primates) for the investigation of endometrial turnover molecular mechanisms. However, Peitz, in an early study of *A. cahirinus* uterine cycle, did not observe menstruation in this species after evaluating 110 animals [5].

The present study provides a comparative and descriptive assessment of uterine cycle in a colony of spiny mice (identified as *A. cahirinus*) at Lomonosov Moscow State University and demonstrates absence of menstrual bleeding along with observation of typical decidual cells within endometrial tissue.

## 2. Results

### 2.1. Vaginal Smears from Spiny Mice Demonstrate Absence of Menstrual Bleeding

**Figure 1:**
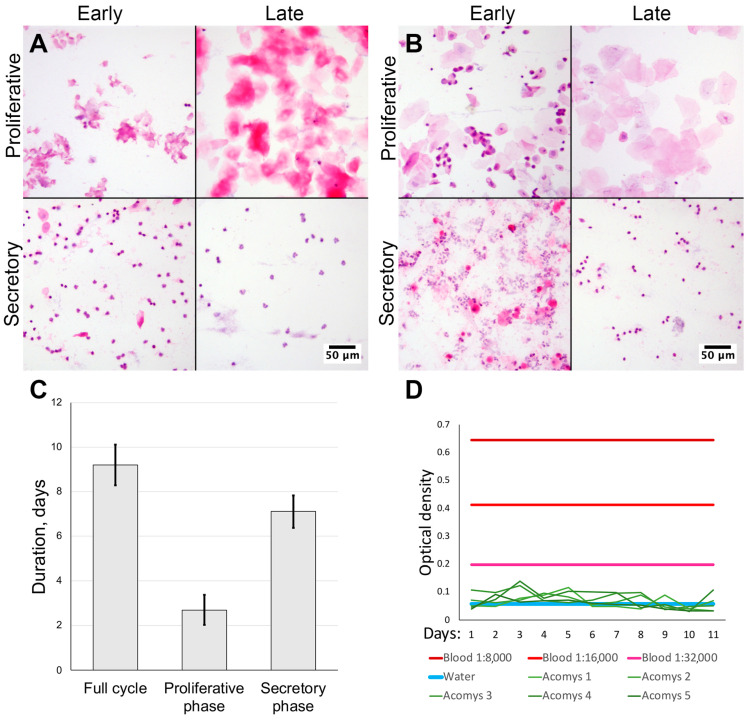
Assessment of cycle duration and blood appearance in vaginal smears from spiny mice. Microscopy of vaginal smears from CD1 strain laboratory mice (**A**) and *Acomys* genus spiny mice (**B**); representative images after staining by hematoxylin and eosin, 200× magnification (brightfield microscopy). Uterine cycle phases duration was determined by smear cytological analysis (**C**). Lack of bleeding and absence of erythrocytes was supported by occult blood assay (**D**) (n = 5).

We obtained vaginal smears from female spiny mice of our colony (n = 10) and CD1 strain laboratory mice (n = 3) stained with hematoxylin and eosin. By examining the smears using microscopy, we estimated the uterine cycle in laboratory mice to be 6.33 ± 0.57 days. During proestrus we observed epithelial cells with nuclei being replaced by anuclear epithelial cells in estrus. In metestrus a mixture of epithelial cells and neutrophiles was observed with neutrophile predominance in diestrus (Figure 1A).

In spiny mice, the estimated duration of uterine cycle was 9.20 ± 0.92 days. Smears in the proliferative phase were similar to those in laboratory mice: initially observed nucleated epithelial cells were followed by anucleated cells (Figure 1A). In the early secretory phase, we observed clustered neutrophiles and numerous typical epithelial cells that turned anucleated by the late secretory phase and disappeared altogether; at this point neutrophiles no longer formed clusters. In spiny mice the duration of the proliferative phase was 2.70 ± 0.67 days, followed by a secretory phase of 7.1 ± 0.74 days (Figure 1A,C). In proportional terms, the proliferative phase comprised approximately 30%, while the secretory phase was about 70% of cycle.

The major finding of this observation was the lack of erythrocytes in vaginal smears from spiny mice in our colony (Figure 1A,B), suggesting the lack of menstrual bleeding that can be detected by smear microscopy.

### 2.2. Lack of Occult Blood in Vaginal Swabs from Spiny Mice

The lack of erythrocytes in vaginal smears from spiny mice contradicted published data [6,7] so we decided to detect blood by another method. We adapted the qualitative occult blood assay used in medical and forensic practice for semi-quantitative detection of occult blood in vaginal swabs from spiny mice. The method has shown the detection of blood diluted with saline at ratios as low as 1:32,000.

Daily vaginal swabs were collected from five female spiny mice (from the same group we used for vaginal smears) for 11 days and assayed for occult blood at endpoint. Assay results in all samples were below the limit of detection and never reached the values of positive control, regardless of the dilution used (Figure 1D). Since the minimal positive control sample had a 1:32,000 dilution, we stopped collection after samples from five sequentially tested spiny mice returned negative results, in order to follow ethical guidelines and avoid further stressful and invasive testing. Thus, using an analytical method, we have validated our microscopy results which indicated a lack of menstrual bleeding in spiny mice.

### 2.3. Decidual Cells Are Present in Spiny Mouse Uterine Stroma During the Late Secretory Phase

Substantial duration of the secretory phase in spiny mouse (Figure 1C) was concordant with data on active luteal phase in this species [6]. Macrophotographs of uteri were obtained from spiny mice in the early (n = 5) and late (n = 5) secretory phases, as well as microphotographs of tissue cross-sections stained by hematoxylin and eosin.

**Figure 2:**
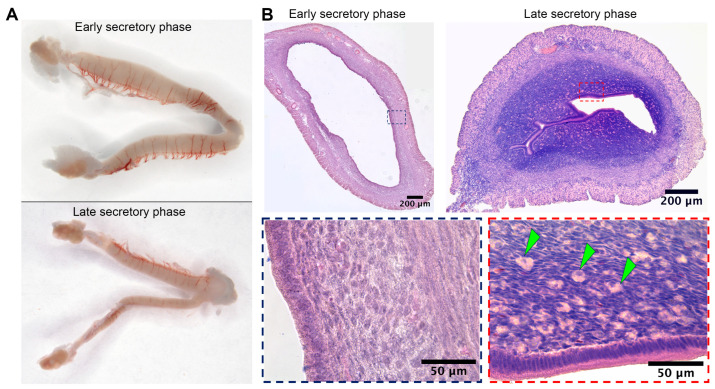
Wholemounts of spiny mice uteri in the early and late secretory phases and evidence for decidua formation in spiny mouse during the late secretory phase. Gross photographs of spiny mouse uteri (**A**) demonstrated minor transverse size increase during early secretory phase, while histology (**B**) demonstrated the presence of large round-shaped decidual cells (arrowhead marks) within endometrium of a spiny mouse exclusively in the late secretory phase; staining by hematoxylin and eosin, 200× magnification (brightfield microscopy).

Gross examination of isolated spiny mouse uteri has shown their increased transverse size in the early secretory phase compared to the late one (Figure 2A). In histological analysis of endometrial stroma during the late secretory phase, we found numerous large round cells showing decidual morphology that clearly distinguished them during microscopy (Figure 2B, arrowhead marks).

**Figure 3:**
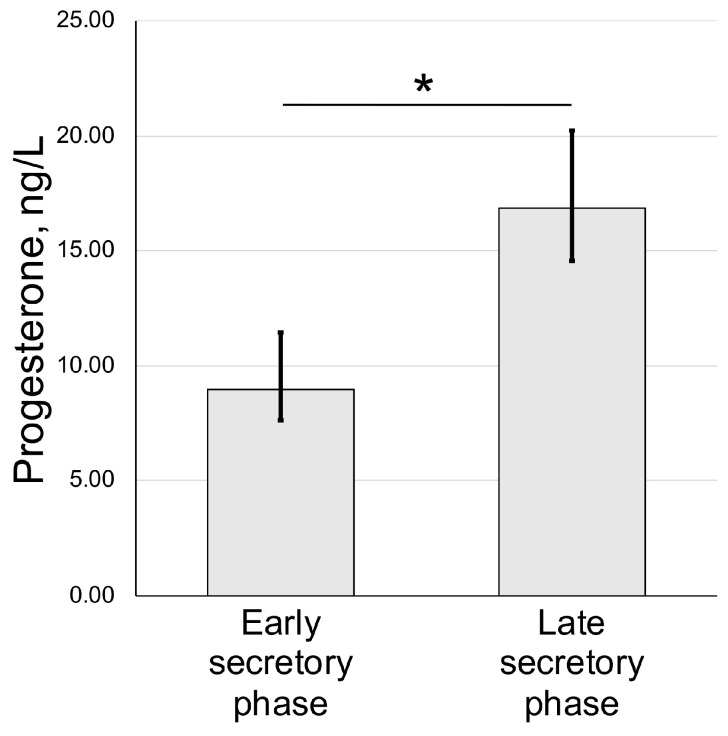
Serum progesterone dynamics in spiny mouse secretory phases of uterine cycle. Enzyme-linked immunoassay results (mean ± S.D.); * *p* < 0.05 Mann–Whitney U-test (n = 5).

We also assayed the concentration of progesterone in blood serum of animals used for uteri harvest and histological assessment. We have detected an increase from 9.33 ± 3.08 nmol/l (early secretory phase) to 17.94 ± 7.88 nmol/l in the late secretory phase (Figure 3).

### 2.4. Structural Traits of Spiny Mouse Endometrium

#### 2.4.1. Epithelial Structures in the Endometrium of Spiny Mouse and CD1 Strain Laboratory Mouse

**Figure 4:**
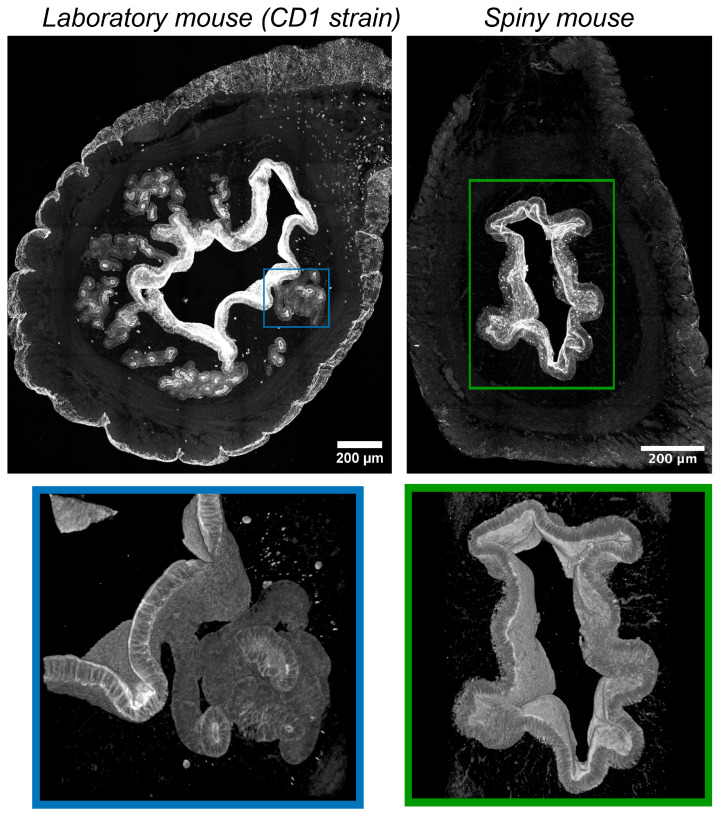
Visualization of epithelial structures in uteri of CD1 strain laboratory mouse and spiny mouse. Confocal microscopy of thick (120 μm) sections after immunolabeling of cytokeratins (markers of epithelial cells); maximum projection along the *z*-axis and 3D reconstruction.

In CD1 mice immunofluorescent labeling of epithelial cells by antibodies vs. cytokeratins detected numerous convoluted glands with complex branched morphology evenly distributed within the endometrium (Figure 4). Glandular lumens had clearly distinguishable boundaries with their mouths opening into the endometrial cavity.

On the contrary, in spiny mice, we detected no glandular structures, while the surface of the endometrium was covered by a layer of epithelial cells. In the late secretory phase, we found numerous “epithelial depressions”: invaginations of the epithelium into the stroma not observed in the early secretory phase. For additional detail, see Supplementary Videos S1 and S2.

#### 2.4.2. Comparison of Nerve Fiber Distribution in the Uteri of Spiny Mouse and CD1 Strain Laboratory Mouse

**Figure 5:**
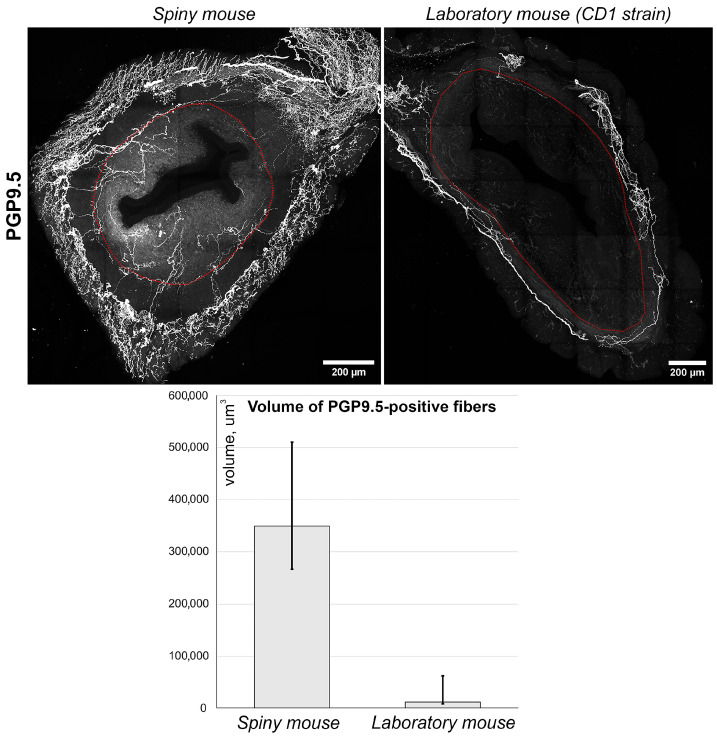
Immunolabeling and quantitative morphometry of nerve fibers within endometrium of CD1 strain laboratory mouse and spiny mouse. Images were obtained by confocal microscopy of 120 μm sections immunolabeled for PGP_9.5 (nerve fiber marker); the red dotted line shows the endometrium/myometrium border. **Graph** represents results of quantitative morphometry of nerve fiber volume in endometrium of laboratory (n = 3) and spiny (n = 12) mice; (*p* < 0.05) Mann–Whitney U-test.

Using anti PGP_9.5 antibodies, we visualized nerve fibers clearly detectable in both species (Figure 5, images). Nerve fibers were localized predominantly in the myometrial layer and only scarce innervation was observed in the endometrium. Furthermore, the density of PGP_9.5 + fibers was visually higher in spiny mice. To obtain comparative data, we performed a semi-quantitative assessment of PGP_9.5 + structures using volumetric morphometry. Generally, the volume of nerve fibers in thick sections of spiny mice uteri was significantly higher (*p* < 0.05) than in CD1 mice (Figure 5, graph).

#### 2.4.3. Comparison of Smooth Muscular Structures of the Uterus of Spiny Mouse and CD1 Strain Laboratory Mouse

**Figure 6:**
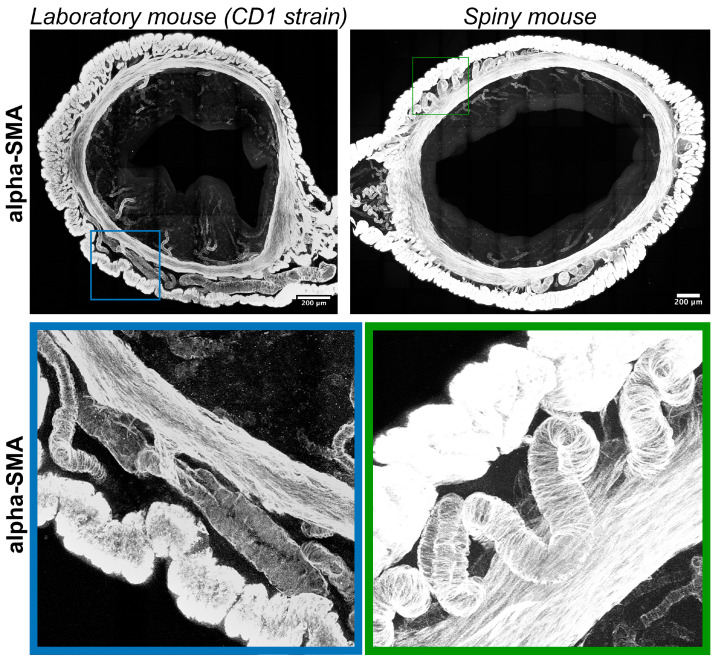
Visualization of smooth-muscle actin in uteri of CD1 strain laboratory mouse and spiny mouse. Confocal microscopy of thick (120 μm) sections labeled with a-smooth muscle actin; maximum projection along the Z-axis and 3D reconstruction.

Immunolabeling for α-smooth muscle actin (α-SMA) visualized transverse layers of smooth muscles in the myometrium with interlayer arteries and arterioles localized in the endometrium. Compared to CD1 strain arteries in spiny mice, myometria had tortuous (spiral) morphology (Figure 6).

#### 2.4.4. Comparison of Uterine Lymphatic Vessels in a Spiny Mouse and CD1 Strain Laboratory Mouse

**Figure 7:**
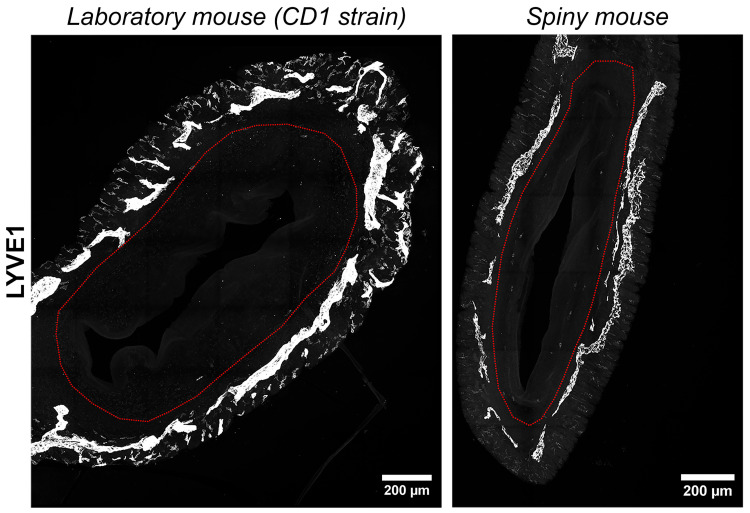
Visualization of lymphatic vasculature in uteri of CD1 strain laboratory mouse and spiny mouse. Confocal microscopy of thick (120 μm) sections labeled with the hyaluronic acid receptor of the lymphatic vessel endothelium (LYVE1), maximum projection; red dotted line indicates the endometrium/myometrium border.

We have visualized lymphatic vasculature by immunofluorescent labeling for hyaluronic acid receptor of the lymphatic vessel endothelium (LYVE1). In both species uterine lymphatic vessels were predominantly localized between the longitudinal and transverse layers of the myometrium without penetration into the endometrium (Figure 7). In general, no significant difference in lymphatic vessel morphology was observed between the species.

## 3. Discussion

Menstrual cycle is confined to a certain set of species, including humans, and evolutionary basis for this strategy remains highly enigmatic [1]. However, the investigation of menstrual cycle molecular patterns is important for several reasons. First, repeated scar-free healing after each shedding of endometrium is an amazing case in humans, indicating conserved ability for regeneration. It also provides targets that can be utilized for interventions inducing similar outcomes in tissues prone to fibrosis [4,8,9]. Second, the understanding of menstrual cycle regulation may unveil targets for treatment of numerous conditions in female reproductive system that cause infertility or hinder pregnancy (e.g., thin endometrium, Asherman syndrome, endometriosis, etc.).

Relevant animal models in this field are scarce, especially murine ones, which are the most feasible for researchers, yet commonly used *M. musculus* strains, characterized by an estrous cycle, do not always fit the task [10]. Recent reports on spiny mouse, particularly its Cairo (also known as *Egyptian* or *Arabian*) species known as *A. cahirinus,* characterized by menstruation [5], triggered interest to this object. This provided a significant pool of data using them as an experimental animal [7,10,11,12,13,14,15,16], while reports on active menstrual bleeding in this animal were contradictory.

Based on our results, we can conclude that the spiny mouse is an interesting research object, and, despite the absence of menstruation in animals from our colony, it shows the ability for spontaneous decidualization (Figure 1 and Figure 2) correlated with an increase in serum progesterone (Figure 3).

Focusing on the first point—the failure to detect menstrual bleeding in spiny mouse—once smear microscopy was not convincing enough, we used a chemical occult blood assay (Figure 1D) to confirm the absence of bleeding. However, our results are concordant with an earlier study by B. Peitz [6] who did not observe menstruation in her colony of *A. cahirinus* after evaluating 110 animals. It should be noted that B. Peitz detected erythrocytes in vaginal smears, but only after the induction of decidual reaction by traumatization.

A putative explanation of this contradiction is the potential existence of subspecies with specific traits. This issue currently under genomic investigation to clarify the diversity within *Acomys,* which have even been given different region-specific names due to the wide distribution of spiny mice. Indeed, as far as we know, the genomic assembly [17] for *A. cahirinus* has yet to be cross-aligned and validated. As a result, researchers in the field lack a reliable reference genome to confirm which animal they are working with.

The impact of stress, known to cause menstrual cycle disturbance in women, is also barely a factor in our case, since spiny mice in our colony were getting regular pregnancies and normal litters, uncommon in human with amenorrhea. Interestingly, female *A. cahirinus* in the colony of Dr. Oleg Gusev’s group [18] at Kazan federal university (Kazan, Russia) have demonstrated vaginal bleedings (personal communication). This basically excluded our first suggestion on potential climate or stress impact on rodents, suggesting instead that we are using two different subspecies of *Acomys* with specific reproductive traits. This partially explains why certain groups may acquire different observations in this genus. Overall, we may have failed to observe bleeding as Bellofiore’s group, while the spontaneous decidualization in spiny mice from our colony supports menstrual turnover of endometrium.

A peculiar finding we specifically address in our work is the observation of well-distinguished decidual cells in the endometrium of spiny mice from our colony (Figure 3). This feature was of interest because in mammals that do not menstruate, decidualization is triggered by embryo implantation into the uterine mucosa. In menstruating ones, decidualization occurs regardless of fertilization under influence of sex hormones, and signs of decidualization were found in the uteri of non-pregnant spiny mice from our colony, suggesting high similarity to humans. Other authors also reported decidualization in this species while none have previously communicate on detection of such cells [7,11].

It should be noted that, since we did not observe prominent bleeding, we need to address the contradiction between obvious decidualization and the lack of menstruation. Our initial focus on blood expulsion through reproductive tract resulted in a limitation that should be the focus of future studies. Specifically, the weight/thickness of the uterine wall as indicators of edema, along with histological observations using longitudinal sections, may detect partial shedding of the endometrium lining. Furthermore, the diversity of *Acomys* colonies may also impact observations; thus, every researcher might require colony-specific tests prior to further investigation.

Structural traits of spiny mouse uteri were investigated in thick (100–120 μm) sections and, to our knowledge, this is the first study providing such data. A major strength of the analysis in thick sections with 3D reconstruction is a certain degree of confidence in detecting the presence/absence of structure and resulting quantitative morphometric data in a volume of tissue.

Two major differences between the spiny mice from our colony and CD1 laboratory mice were related to glandular structures and the innervation of endometrium (Figure 4 and Figure 5). Interspecies differences in smooth-muscle cell-bearing structures (muscular layer, arteries and arterioles) and lymphatic vessels were less pronounced (Figure 6 and Figure 7).

We were much surprised by the absence of glandular structures in the stroma of spiny mice endometrium contrasting their abundance in CD1 mouse strain. It is important to note that human endometrium has prominent well-developed glands [7]. Using fluorescent microscopy of thick cleared samples of the endometrium, a tubular network of glandular epithelium, the so-called “rhizome”, was discovered, from which seemingly separate simple tubular glands rise to the epithelium, lining the uterine cavity [19]. According to our data, the glands in CD1 mice are also not plainly tubular, but branched. The absence of glands in spiny mice, on the one hand, may question the degree of similarity to humans, but, on the other hand, it suggests the role of stroma in endometrium growth.

This feature is of importance since the stromal compartment of endometrium drives its growth under the action of estrogens [20,21,22]. In murine species research attention is generally drawn to epithelial cells due to their estrogen-dependent proliferation, while *A. cahirinus* may be a feasible rodent model for understanding the mechanisms of endometrial stroma renewal and growth.

Another trait of spiny mice was higher density of nerve fibers compared to CD1 mouse strain, which should be taken into account when using them for experimental studies. Generally, the role of nerve fibers in the regulation of endometrial function has been poorly studied. It is only known that one of the possible diagnostic tests for minimal to mild endometriosis is the evaluation of nerve density in the eutopic endometrium [23]. Overall, this stresses the importance of neural regulation of endometrial functions, and *A. cahirinus* is a rodent species with a more pronounced dense endometrium innervation (Figure 5) compared to *M. musculus.*

In humans, spiral arteries are found in the endometrial functional layer, and desquamation is preceded by their constriction [24]. In our observations spiral arteries were not detected in both species, while only in *A. cahirinus* similar vascular structures were penetrating into the myometrial layer (Figure 6). The significance of this morphological feature is yet to be investigated. Unlike arteries, the appearance of lymphatic vessels did not show any significant differences (Figure 7).

One may be interested in how these features may impact regenerative capacity and menstrual cycle turnover in spiny mice. Unfortunately, recent data in the field remains limited; thus, our data indicates further directions of investigation—namely, the roles of the peripheral nervous system, regulation of decidualization, and the role of spiral artery in menstruation—once its hallmarks (bleeding and tissue discharge) are detected in future studies.

## 4. Materials and Methods

### 4.1. Animal Strains and Husbandry

Spiny mice (identified as *Acomys cahirinus*) were obtained from the Moscow Zoo (Moscow, Russia), and a colony was established at Lomonosov Moscow State University Faculty of Medicine (Moscow, Russia). Spiny mice were housed in groups of 5–6 per cage. Cages were lined with wood shavings and supplemented with cardboard tunnel structures and wooden houses. Food (chow pellets, special food, and water) was available ad libitum, and fresh vegetables were added weekly at a rate of up to 50 g per cage, including carrots and celery. Temperature was maintained between 25 and 27 °C and humidity at 30–40%, with a 12 h light/dark cycle (lights on at 7 am).

CD1 laboratory mice were obtained from Laboratory Animal Nursery “Pushchino” (Pushchino, Russia) and housed in cages of 4–6 individuals, with ad libitum access to water and standard mouse food at 20–22 °C and humidity at 40–60%.

Animal manipulations and euthanasia procedures were performed in compliance with National and European Union regulations and were approved by the Institutional (Ethics Board for Animal Care) Animal Care and Use Committee (Lomonosov Moscow State University; permit 205-G; 27 March 2025).

### 4.2. Design of Experimental Studies

General concept was based on comparative analysis of uterine cycles in female wild-type spiny mouse and CD1 mouse strain (*Mus musculus*). For this purpose, vaginal swabs with subsequent staining were used to determine the timespan of the cycle, and occult blood tests were used to detect blood in the vaginal discharge. To assess the level of progesterone, blood serum from the heart cavity was used for enzyme immunoassay. Eventually we obtained uterine samples for histological staining and immunofluorescent labeling followed by light or fluorescent confocal microscopy to analyze the morphological dynamics.

### 4.3. Vaginal Smear Collection and Analysis

Vaginal smears were collected daily from 10 spiny mice and 3 female CD1 mice aged 3–4 month and 3–4 weeks, respectively, using sterile saline. Female spiny mice were placed supine with a small towel gently wrapped around the neck and shoulders due to fragile connective tissue and tendency for the skin to tear. The vulva was moistened with an unscented water-based lubricant and approximately 50 μL of saline was drawn to a 200 μL disposable plastic tip. The tip was inserted into the vaginal canal to a depth of no more than 8 mm well before contact with the cervix and saline was injected into the vagina twice. The solution was withdrawn back into the pipette, applied to a glass slide and allowed to dry for 5 min at room temperature. Smears were fixed by placing the slides successively in 70% and 96% ethyl alcohol for 5 min each.

Fixed samples were stained with aqueous hematoxylin and eosin (BioVitrum, Moscow, Russia) as follows. Fixed slides were washed in PBS for 30 s to 1 min. Then they were placed in hematoxylin solution for 2 min, washed by tap water, and placed in eosin solution for 15 min. Slides were then washed three times with tap water to remove excess dye. Stained slides were gradually dehydrated in several ethanol solutions (70% and 96%), air-dried, and mounted with Dako Mounting Medium (Agilent, Santa Clara, CA, USA) under coverslips. Cytological studies of the stained smears from both spiny and CD1 mice were made at 100- and 200-fold magnification using light microscope (Leica Microsystems, Wetzlar, Germany).

### 4.4. Occult Blood Test

To detect blood in vaginal smears from 5 of 10 spiny mice, a modified Gregersen test (benzidine test) was used to assay occult blood in the material. The peripheral blood as a positive control was obtained from 2 more spiny mice. Before the test, vaginal smears and spiny mouse peripheral blood were frozen five times in liquid nitrogen and thawed at 37°C for lysis. In a 96-well plate, 45 μL of TMB (3,3′, 5,5′-tetramethylbenzidine), 45 μL of 40% acetic acid, and 10 μL of 10% H_2_O_2_ were mixed in each well. Then 50 μL of vaginal smear material was added, with diluted spiny mouse blood (from 1:8000 to 1:32,000) as a positive control and water as a negative control. After 30 min of incubation, the color change in the samples in the wells was visually determined (in the case of a positive result—the presence of hemoglobin—the color of the solution changed from colorless to yellow; the color intensity increased in direct proportion to the amount of hemoglobin), and the change in the optical density of the solution was determined using a spectrophotometer (Perkin-Elmer, Shelton, CT, USA) at λ = 450 nm.

### 4.5. Animal Euthanasia and Sample Harvest

The animals (spiny mice or CD1 mice) were sacrificed by CO_2_ asphyxiation, and cardiac puncture was performed to draw 0.5–1.0 mL of blood from each animal. Blood samples were centrifuged in cooled (4°C) Eppendorf 5415R centrifuge (Eppendorf, Hamburg, Germany) for 20 min at 2000 rpm, and obtained supernatant serum samples were collected and frozen at −80°C for further analysis. After blood collection, the abdomen of sacrificed mouse was cleaned by 70% alcohol wipe, and about 10 mL of 10% formalin solution (for spiny mice) or about 5 mL of 10% formalin (for CD1 mice) was injected into the abdominal cavity by a 15 mL syringe to fix the uterus for 10 min. Then, formalin was removed from the abdominal cavity, and the collection of uteri was performed. Obtained samples were placed in 14 mL of 10% formalin solution, followed by taking macro images of organs using a stereomicroscope (Nikon, Tokyo, Japan)

After 2 days in formalin, the organs were dissected from residual fat and cut into the following parts: the uterus was divided into 2 horns (I and II) with 4 equal parts from each horn going from the base of the uterus to the ovaries. The numbered parts of the uteri were divided for either immunofluorescent labeling or routine histology stains.

### 4.6. Serum Progesterone Assays

Quantitative analysis of progesterone in the serum of spiny mice was carried out using «ImmunoFA-Progesterone» enzyme-linked immunosorbent assay (ELISA) kits (Immunoteks, Moscow, Russia). Prior to the experiment, 25 μL of calibration samples, control serum or test blood sera with 0.1 mL of conjugate was first added to the wells of a 96-well plate and incubated for 1 h at 37°C. After washing, 0.1 mL of substrate solution was added to the wells and incubated for 10–15 min at 37°C, followed by 0.1 mL of stop reagent. A spectrophotometer (Perkin-Elmer, Shelton, CT, USA) was used to measure the optical density at λ = 450 nm.

### 4.7. Routine Histology and Hematoxylin–Eosin Staining

For histological staining, the uteri and ovaries were initially placed in a 30% sucrose solution for 24 h and then frozen in liquid nitrogen in Tissue-tek^®^ OCT compound (Sakura Finetek, Torrance, CA, USA) in cryomolds.

Sections with a thickness of 15 μm were prepared on a cryotome (Leica Microsystems, Wetzlar, Germany) and attached to glass slides (Epredia, Kalamazoo, MI, USA), and dried at room temperature for 24 h. The sections were stained with hematoxylin and eosin (5 min with intermediate rinse in tap water) and gradually dehydrated in ethanol solutions (40% and 70%). Aqua-Poly/Mount medium (Polysciences, Warrington, PA, USA) was used to embed the preparations under coverslips.

Microimages were obtained using a microscope with constant illumination at 200× magnification and analyzed using Fiji software (v. 1.54r, NIH, Bethesda, MD, USA).

### 4.8. Immunofluorescent Labeling and Confocal Microscopy

Thick (100–120 μm) sections of isolated uteri were prepared using a Leica 1200s vibrotome (Leica Microsystems, Wetzlar, Germany) after incubation for 12 h in 50% tetrahydrofuran (Reactivetorg, Moscow, Russia). Immunofluorescent labeling was performed directly in 0.5 mL tubes. The antibody solution contained PBS, 0.1% Triton X−100, 2% goat serum (Thermo Fisher Scientific, Waltham, MA, USA). The samples were incubated for 16 h at 37 °C with antibodies to α-SMA (ab5694, Abcam, Cambridge, UK), LYVE1 (ab14917), PGP_9.5 (ab108986) at a 1:100 dilution, and CK (ab9377) at a 1:50 dilution. After washing from the primary antibodies by PBS containing 0.1%, Triton incubation with the corresponding rabbit secondary antibodies (Invitrogen, Waltham, MA, USA) was performed for 16 h at 37°C. Final washing was performed 3 times for 1 h. The nuclei were labeled with DAPI (Sigma-Aldrich, Burlington, MA, USA) for 8 h (1 μg/mL) in parallel with clearing of the samples according to the EZ clear protocol [25]. Upon completion of the labeling and clearing, the samples were placed in the same clearing solution with metal spacers, and were analyzed by confocal microscopy using a Leica TCS SP5 (Leica Microsystems, Wetzlar, Germany). The obtained images were processed and analyzed with Fiji software (NIH, Bethesda, MD, USA).

### 4.9. Statistical Analysis

Data processing and statistical analysis were performed in Excel and StatPlus v.8 (AnalystSoft Inc, Tampa, FL, USA) software. Comparison of independent groups was performed using the Mann–Whitney U test. A 0.05 significance level was applied.

## 5. Conclusions

Overall, our results highlight the need for further investigation of the spiny mouse as an animal model of menstruation.

Female *Acomys* genus mice (identified as *A.cahirinus*) from a colony located at Lomonosov Moscow State University after acquisition from the Moscow Zoo demonstrated spontaneous decidualization without uterine bleeding, suggesting a lack of typical menstruation. Endometrium glands present in humans were not observed in spiny mouse uteri, while endometrial innervation was more prominent than in CD1 house mouse (*M. musculus*). These features make spiny mouse a feasible object to study the role of stromal cells and uterus innervation in regulation of menstrual cycle and endometrium turnover or regeneration after eventual shedding (e.g., after pregnancy and pups’ birth).

The diversity of *Acomys* spiny mice menstrual cycles in different reports warrants establishment of molecular identification tools and harmonization of species identity protocols used in different colonies. This will provide a sustainable basis for future comparative studies investigating physiological mechanisms of menstruation.

## Data Availability

Data are contained within the article.

## Supplementary Materials

The following supporting information can be downloaded at https://www.mdpi.com/article/10.3390/biology14101365/s1, Video S1: Confocal microscopy 3D reconstruction of spiny mouse endometrium after immunolabeling for cytokeratins; Video S2: Confocal microscopy 3D reconstruction of laboratory mouse endometrium after immunolabeling for cytokeratins.

## Abbreviations

The following abbreviations are used in this manuscript:

CD	Cluster of differentiation
DAPI	4′,6-diamidino-2-phenylindole
ELISA	Enzyme-linked immunosorbent assay
TMB	TMB (3,3′, 5,5′-tetramethylbenzidine),
